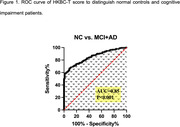# Norming and standardization of Hong Kong Brief Cognitive Test and diagnostic study on the effectiveness of screening patients with cognitive impairment

**DOI:** 10.1002/alz.092238

**Published:** 2025-01-03

**Authors:** Yaonan Zheng, Helen Y. L. Chan, Nan Li, Jiahui Zhu, Minyue Pei, Xin Yu, Chuan Shi, Huali Wang

**Affiliations:** ^1^ Peking University Institute of Mental Health (Sixth Hospital), Beijing China; ^2^ The Chinese University of Hong Kong, Hong Kong China; ^3^ Clinical Epidemiology Research Center of Peking University Third Hospital, Beijing China

## Abstract

**Background:**

The Hong Kong Brief Cognitive Test (HKBC) has solved the problems of limited population, insufficient coverage of cognitive domains and copyright limitation of previously used cognitive function test tools, which has a preliminary research basis for promotion. To make HKBC more widely used, it is necessary to collect normative data from different regions to make it have reference standards.

**Method:**

From September 2019 to December 2021, 2312 aged 40 years and older people were recruited from different regions of China as norm construction participants. The diagnostic study included 93 normal elderly patients and 224 patients with cognitive impairment (including 156 patients with mild cognitive impairment and 68 patients with Alzheimer’s disease). Multinomial regression model was used to analyze the correlation between HKBC score and age, sex, living area and education level. The norm of HKBC total score and T score (HKBC‐T) were constructed. Based on the receiver operating characteristic curve, the AUC, sensitivity, specificity and Youden index of HKBC‐T were calculated to determine the optimal cutoff value of cognitive impairment.

**Result:**

The results showed that HKBC score was negatively correlated with age (SE = 0.007, β = ‐0.067, P < 0.001) and positive correlation with education (SE = 0.016, β = 0.280, P < 0.001), no significant association with gender and living area (p > 0.05). According to different ages and education levels, norm of HKBC total score and HKBC‐T were constructed, and the conversion equation of HKBC‐T was calculated. The results of HKBC‐T showed a good AUC value of 0.85 in distinguishing patients with cognitive impairment from normal elderly people. The optimal cutoff value for the diagnosis of cognitive impairment by the HKBC‐T was 46 points (sensitivity = 0.914, specificity = 0.667).

**Conclusion:**

The HKBC score is influenced by age, living area and education level, which provides a scientific basis for the development of HKBC norm model. The results of the diagnostic study showed that the optimal cutoff value of the HKBC‐T was 46 when distinguishing patients with cognitive impairment from the normal elderly.